# Differential Analysis of Genetic, Epigenetic, and Cytogenetic Abnormalities in AML

**DOI:** 10.1155/2017/2913648

**Published:** 2017-06-20

**Authors:** Mirazul Islam, Zahurin Mohamed, Yassen Assenov

**Affiliations:** ^1^Pharmacogenomics Lab, Department of Pharmacology, Faculty of Medicine, University of Malaya, Kuala Lumpur, Malaysia; ^2^Computational Epigenomics Group, Division of Epigenomics and Cancer Risk Factor, German Cancer Research Center, Heidelberg, Germany; ^3^Department of Medical Oncology, Dana-Farber Cancer Institute, Boston, MA, USA

## Abstract

Acute myeloid leukemia (AML) is a haematological malignancy characterized by the excessive proliferation of immature myeloid cells coupled with impaired differentiation. Many AML cases have been reported without any known cytogenetic abnormalities and carry no mutation in known AML-associated driver genes. In this study, 200 AML cases were selected from a publicly available cohort and differentially analyzed for genetic, epigenetic, and cytogenetic abnormalities. Three genes (FLT3, DNMT3A, and NPMc) are found to be predominantly mutated. We identified several aberrations to be associated with genome-wide methylation changes. These include Del (5q), T (15; 17), and NPMc mutations. Four aberrations—Del (5q), T (15; 17), T (9; 22), and T (9; 11)—are significantly associated with patient survival. Del (5q)-positive patients have an average survival of less than 1 year, whereas T (15; 17)-positive patients have a significantly better prognosis. Combining the methylation and mutation data reveals three distinct patient groups and four clusters of genes. We speculate that combined signatures have the better potential to be used for subclassification of AML, complementing cytogenetic signatures. A larger sample cohort and further investigation of the effects observed in this study are required to enable the clinical application of our patient classification aided by DNA methylation.

## 1. Introduction

AML is a haematological disorder characterized by excessive proliferation of undifferentiated myeloid cells [1] in the bone marrow that infiltrate the liver, spleen, lymph node, and circulating blood [2]. This cancer type progresses rapidly and is relatively fatal due to acquired genetic and/or cytogenetic aberrations. In 2014, AML was the most common type of leukemia diagnosed and accounted for 1.78% of predicted cancer deaths in the United States [3]. Overall, the 5-year survival rate varies drastically based on the cytogenetic risk classification: 55%, 24%, and 5% overall survival for favourable, intermediate, and adverse risk group patients, respectively [4]. Relapse is the major reason for a poor survival rate as it occurs in up to 80% of AML patients [5]. Almost 50% of all AML cases belonging to the intermediate risk group have been reported to lack any cytogenetic abnormalities [6]. Furthermore, a significant proportion of the patients carry no reported genetic mutations in any known AML-associated driver genes [7, 8]. These findings clearly indicate that there are other elements predisposing to and driving the disease in the case of cytogenetically normal AML (CN-AML).

It has already been reported that epigenetic modifications are involved in the regulation of haematopoietic development [9, 10]. DNA methyltransferases and histone methyltransferases are well-known epigenetic modifiers that contribute to cellular identity through regulation of gene expression in myeloid progenitor lineages. They are among the frequently mutated group of genes in AML [11, 12] which suggests that epigenetic modification could be a causative agent for disease progression and relapse in CN-AML. HOX genes are often hypomethylated in CN-AML, whereas DNMT3A tends to be mutated [13]. Mutation of other epigenetic regulators, such as TET2 [14], MLL [15], IDH1/2 [16], FLT3 [11], NPM1 [11], RUNX1 [17], and ASXL1 [18], has been associated with unfavourable clinical outcomes for AML patients [7]. Therefore, mutations in epigenetic modifiers as well as alterations of genome-wide methylation patterns in AML imply epigenetic deregulations as one of the fundamental causal agents in AML pathogenesis.

AML is a biologically heterogeneous and complex malignant disorder, and no single causative agent has yet been identified which is capable of oncogenic transformation alone. The overwhelming evidence suggests a complex interplay of genetic events contributing to AML pathogenesis [11]. From the biological point of view, the cancer phenotype is likely directly associated with gene expression, which can be potentially driven by genetic, epigenetic, and cytogenetic changes. In order to explore the pathophysiology of this disease, integration of genetic, epigenetic, and cytogenetic data and differential analysis are required. The combination of information on every single molecular parameter into phenotypic signatures would be a robust classifier in disease diagnosis and risk stratification in AML [19–21]. Disease prognosis and classification of AML are important for physicians to decide particular treatment protocol in order to avoid minimal residual disease and risk of relapse [22]. None of the two well-known classifications (French-American-British [23] and World Health Organization [24]) integrate epigenetic signatures with genetic and cytogenetic ones. To facilitate a molecular pathophysiological study of AML, The Cancer Genome Atlas (TCGA) research network has generated a huge amount of data that are publicly available for further analysis and interpretation of AML pathogenesis, classification, and risk stratification [11]. TCGA is providing the most comprehensive molecular and clinical data on over 33 different tumor types of 10000 cancer patients [25]. Although TCGA has made many important discoveries and published more than 20 marker papers, still remarkable opportunities exist to analyze the data using novel methods and strategies from dynamic viewpoints.

For this study, we used the data available in TCGA under study code LAML and differentially analyzed using a carefully selected set of online and offline platforms. For the comprehensive analysis of differential DNA methylation, we used specifically RnBeads [26]. In order to integrate genome-wide methylation and mutation data with cytogenetic data, we developed custom R scripts analyzing every single event and combining events. We also used circos, ingenuity pathway analysis (IPA) database, and other platforms to organize and visualize the data. We used hierarchical clustering and heatmaps to identify and visualize distinct groups of AML patients and genes. Further, we used a large collection of diagnostic and phenotypic traits to calculate individual and composite risk factors and presented them as Kaplan-Meier curves. Our integration strategy provides useful insight into disease subtype identification, risk measurement, and detection of clinical prognostic markers.

## 2. Materials and Methods

### 2.1. Data Source and Software

We used TCGA (https://tcga-data.nci.nih.gov/tcga/) data repositories as our primary data source for this study. To analyze the AML data generated by TCGA, we directly accessed and downloaded the raw data, using the “Data Matrix” tool provided by TCGA Data Portal. We also downloaded clinical, gene expression, and DNA methylation data of all available AML patients. In total, we found 200 patients having clinical and gene expression data and 194 DNA methylation data (date of download: August 15, 2015). The available data type for DNA methylation analysis was Illumina Infinium HumanMethylation450 BeadChip (450 K microarray). We used human genome (UCSC hg19) as reference. Gene definitions were based on Ensembl release 75.

Data analysis was performed using the R platform (http://www.r-project.org/, version 3.1.3) and a collection of R/Bioconductor packages. We used RnBeads (http://rnbeads.mpi-inf.mpg.de/) to analyze and visualize DNA methylation data. RnBeads is a software tool written in the R programming language for large-scale analysis and interpretation of genome-wide datasets, particularly epigenomic data (bisulfite sequencing and Infinium microarrays) with a user-friendly and customizable analysis pipeline. It provides self-configuring workflow at data input, quality control, preprocessing, and tracking and table stages. We inspected the output, in particular at the exploratory analysis and differential methylation analysis stages, available as interactive hypertext report with high-quality figures and tables [26].

### 2.2. Analysis and Visualization

The analysis presented here can be broadly separated into clinical and molecular analysis. Later on, we combined molecular and clinical data to study the survival proportion. In case of clinical data, we uploaded the preprocessed txt file to RStudio and analyzed every column of available patient information and characteristics. The distribution of all the available genetic and cytogenetic abnormalities reported in clinical data table is visualized using “OncoPrint” that can show overlapping abnormalities, if any, across the patient cohort. For multivariate analysis and low-dimensional representation of AML dataset, we applied principal component analysis (PCA). Later, we run “Wilcoxon rank sum test” for the first eight principal components (PC) against each trait to identify significant association between traits. Multidimensional scaling (MDS) was tested after performing Kruskal's nonmetric test. It is important to note that in many cases, patient clinical data is incomplete. Although we analyzed all available data, a large number of patients' information was missing in the TCGA data file that presented separately and some information was not relevant to this study. We also applied various techniques for batch effect detection, as implemented in RnBeads. We used “heatmap.2” for unsupervised hierarchical clustering of samples and genes in a heatmap. Differentially methylated sites and regions were identified using limma tests and Fisher's method for the combination of *p* values. We used a scatter plot and volcano plot for the visualization of differential methylation with a false discovery rate- (FDR-) adjusted *p* value < 0.05. Enrichment analysis was conducted for overrepresented or underrepresented gene sets using Gene Ontology (GO) terms and presented as word clouds. We analyzed the gene ontology, functional genes, and transcriptomes using the IPA (Ingenuity Systems, Mountain View, CA). The distribution of hypermethylated gene promoters in AML cohort is visualized by a histogram, and highly common genes are presented as a circos plot with corresponding chromosome. We analyzed gene mutations in the patient cohort and presented as a histogram. In the case of survival analysis, we combined available data to draw Kaplan-Meier curves and corrected for the effect of age and gender, adjusted the resulting *p* values using the Benjamini-Hochberg method, and applied a significant threshold of 0.05. We used the survival interval from the date of diagnosis until the date of death or last follow-up.

Sample annotation and DNA methylation data analysis was performed in a systematic way in this study. Combined differential DNA methylation at individual CpGs as well as extended genomic regions increased statistical power, interpretability, and reproducibility [27, 28]. In case of differential DNA methylation analysis, we considered not only statistical significance but also biological significance of the output.

## 3. Results

### 3.1. Characteristics of the Patient Cohort and Clinical Testing

In the primary study cohort, a total of 200 AML patients were listed in the TCGA database. The mean age at diagnosis was 55.50 years; 109 (54.5%) patients were male and 91 (45.5%) female. The majority of the patients were white American (91.50%) and non-Hispanic (97%). Only 106 patients were cytogenetically abnormal. Overall genetic abnormalities were around 80% in the cohort, and patients with both genetic and cytogenetic abnormalities were more prone to death compared to those with single-type abnormality. Detected genetic abnormalities were higher than cytogenetic abnormalities. Additional information, such as average blood cell counts, is shown in Table 1.

Cytogenetic tests were performed for almost every patient (196), and fluorescence in situ hybridization (FISH) test was performed for 155 patients. Only three patients showed previous other haematological disorders, and 14 patients showed other malignancies. Neoadjuvant treatment was prescribed to 24.50% of the patients. The vast majority of the patients (79.5%) had never been exposed to leukemogenic agents before; all-trans retinoic acid- (ATRA) induced apoptosis was reported for only 4 patients. Exposure data was missing for 37 patients. The number of FISH test-positive patients was equal to that of FISH test-negative patients (78), and available data was not found for 44 patients. Molecular abnormalities were detected for only 38 patients, and data was not available for 112 patients. Supplementary Figure S1 available online at https://doi.org/10.1155/2017/2913648 summarizes the clinical tests in the form of bar plot.

### 3.2. Genetic and Cytogenetic Abnormalities

**Figure 1:**
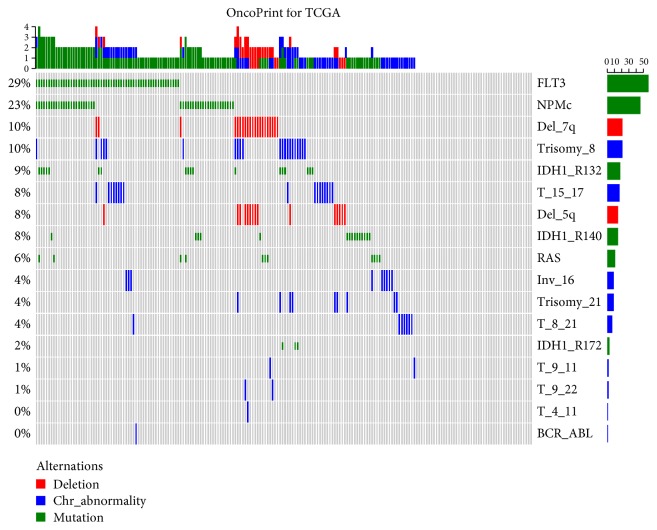
Distribution of genetic and cytogenetic abnormalities in AML. FLT3 and NPMc mutations are the most common abnormalities in AML patients. Many patients have multiple abnormalities. More than 50% of NPMc-positive patients are also FLT3 positive. Gray color represents either negative or unknown.

Almost 30% (*n* = 58) of the patients showed FLT3 mutation (Figure 1()), whereas only one patient showed BCR-ABL fusion and T (8; 21). The second highest mutation rate (23%) was found for NPMc. More than 50% of NPMc-positive patients were also FLT3 positive, and 21 patients were positive for both trisomy 8 and Del (7q). IDH1 R132-positive and T (15; 17)-positive patients were only 18 (9%); IDH1 R140-positive and Del (5q)-positive patients were 15 (7.50%), and activating RAS was reported for only 5.5% of the patients. The rest of the genetic and cytogenetic abnormalities were reported in <5% of the patients. Unknown cytogenetic abnormalities were significantly higher than unknown genetic abnormalities. The highest negative test was reported for IDH1 R172 (96.5%). One notable observation in the clinical data file was that the patients exhibiting multiple abnormalities tended to have shorter survival time. Statistical tests were not significant for many reported abnormalities mostly due to small positive sample size.

### 3.3. Methylation Profiles

**Figure 2:**
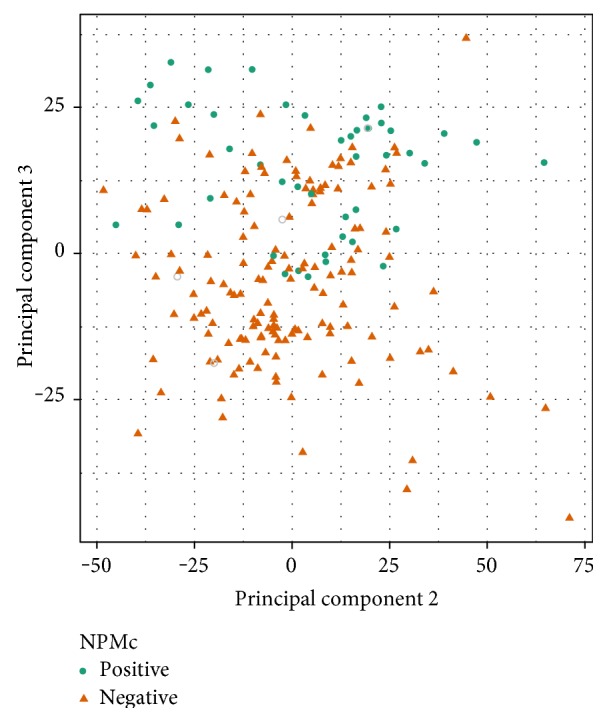
Low-dimensional representation of AML dataset. Scatter plot shows coordinates of NPMc traits on principal components (PC). Gray circles represent missing values. PC2 and PC3 show better separation of NPMc-positive and NPMc-negative patients (positive in upper and negative in lower) than PC1 and PC2 (not shown). PC3 is highly significant for NPMc (Wilcoxon rank sum test, *p* = 5.1*E* − 13).

RnBeads implies two methods for visual inspection of methylation datasets: multidimensional scaling (MDS) and principal component analysis (PCA). After ignoring incomplete features due to missing values, 439751 CpGs were used for MDS and 436441 for PCA. Similarly, promoters used for MDS were 28642 and PCA were 28547. A gene promoter was defined as the region spanning 1500 bases upstream and 500 bases downstream of the gene's transcription start site. The mean number of interrogated probe sites (CpG sites) per promoter is 10 in the dataset. In case multiple CpG sites lie in a particular promoter, we assigned the average methylation (beta) value as the overall methylation level of the promoter. The scatter plot on Figure 2() shows the sample coordinates of the second and third principal components for NPMc. NPMc-positive samples occupy the upper region of the plot, and negative samples are located in the lower region of the plot. Only one sample does not conform to this separation. Although there are some homogenous samples, two groups are clearly clustered in the picture at the top and bottom sides. Second and third principal components (PC) show the strongest association with traits. PC2 and PC3 together can explain around 55% of the total variance, as seen in the cumulative distribution functions. PCA and MDS for FLT3 are shown in Figure S2. The first eight PC together can explain more than 99% of total variance. The Wilcoxon rank sum test shows that PC1 is not significant for any traits except IDH1 R140 (Figure S3).

### 3.4. Batch Effects

Different properties of the dataset were tested for significant associations. All genetic and cytogenetic traits were tested against principal components (Figure S3) as well as between traits (Figure S4). Two statistical tests were performed: Wilcoxon rank sum test and Fisher's exact test where significant *p* values were less than 0.01. In case of association between traits, Del (5q) and Del (7q) were more associated than other genetic traits and cytogenetic traits were more associated than genetic traits. Many association tests could not produce reliable results due to insufficient sample size.

### 3.5. Regional Sites and Methylation Value Distribution in the Whole Genome

**Figure 3:**
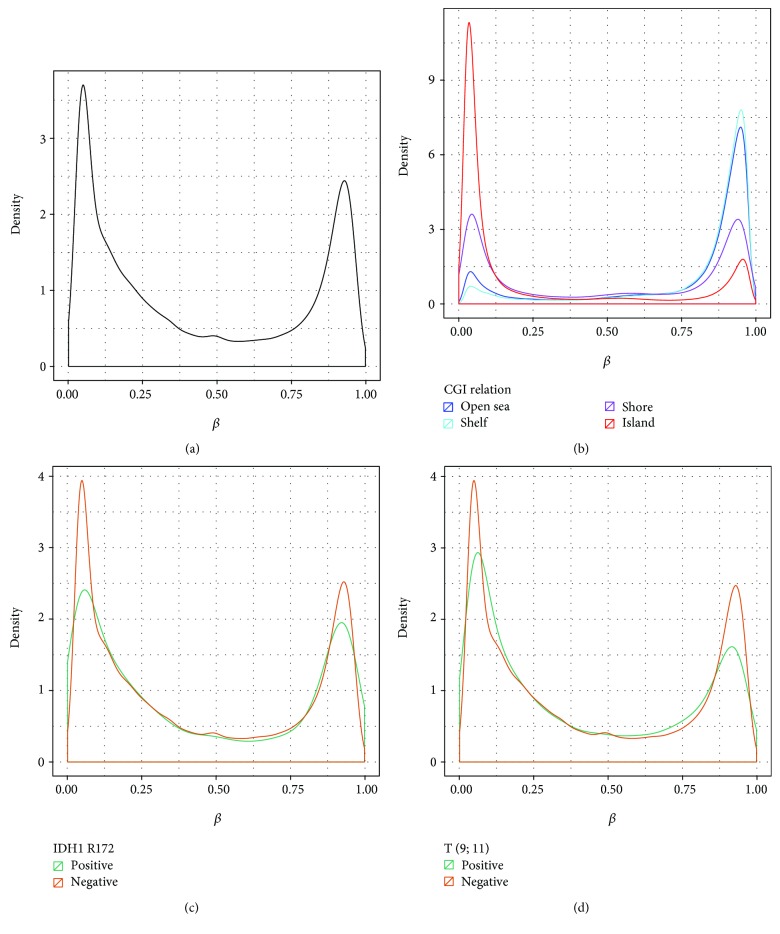
Methylation distribution in genomes. (a) Methylation distribution (beta value) across all promoters that follow bimodal distribution pattern. (b) Methylation value density estimation in all samples at different CpG region. (c) IDH1 R172-positive and IDH1 R172-negative patient's promoter methylation distribution. Negative patient distribution is similar with (a), but positive patients show lower density. (d) T (9; 11)-positive and T (9; 11)-negative patient's promoter methylation distribution, and here, also positive patients show lower density.

After annotation and necessary adjustment, 28642 promoters with available methylation data were identified in the TCGA dataset. Total samples were divided based on abnormality types (genetic and cytogenetic), and among each group, there were positive and negative patients. The average number of CpG sites per promoter was approximately 10. It is worth noting that the reported promoter methylation values are based predominantly on probes located very close to the transcription start site of the corresponding gene. The overall promoter methylation pattern is similar to the genome-wide CpG methylation distribution (Figure 3(a)); that is, it is bimodal. IDH1 R172 and T (9; 11) seem to have an effect on the methylome in the sense that the distributions of promoter methylation for the positive and negative patients differ (Figures 3(c) and 3(d)). This is also the case for other aberrations: FLT3, IDH1 R140, trisomy 8, trisomy 21, and Inv (16) (data not shown). Not suprisingly, we found very high levels of methylation at shelf (2–4 kb from CGI) and then open sea (>4 kb from CGI) regions and much lower at shores (up to 2 kb from CGI) and islands (Figure 3(b)).

### 3.6. Clustering of Samples

**Figure 4:**
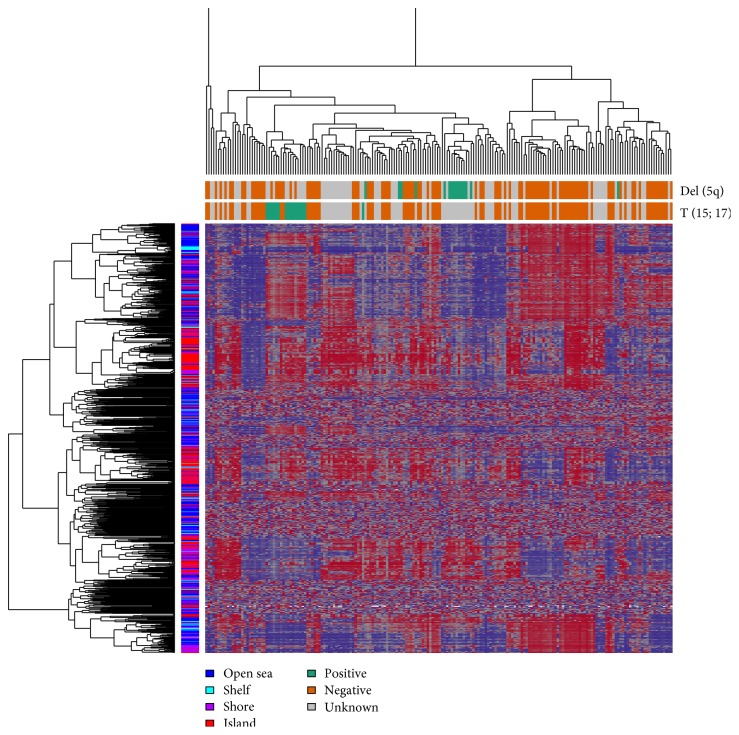
Hierarchical clustering of AML samples. Heatmap displayed only sites with the highest variance across all samples and complete linkage strategy. *x*-axis represents the number of patients, and *y*-axis represents different CpG regions. Gray color in across *x*-axis side bar represents unknown for particular trait. Color key represents beta value (red means hypomethylated and blue means hypermethylated). Del (5q)-positive patients show mostly hypermethylation across the genomic regions. T (15; 17)-positive patients show hypomethylation across the genomic regions.

We had clustered samples hierarchically based on methylation values using correlation-based distance metric and visualized them as heatmaps. Only 2 traits show good clustering: Del (5q) and T (15; 17). The Del (5q)-positive group showed hypermethylation, whereas the T (15; 17)-positive group showed hypomethylation (Figure 4). It is difficult to determine whether the lack of association between other traits and the identified methylation-based patient subgroups (data not shown) can be attributed to the heterogeneity of the disease or the incomplete annotation.

### 3.7. Differential Methylation

**Figure 5:**
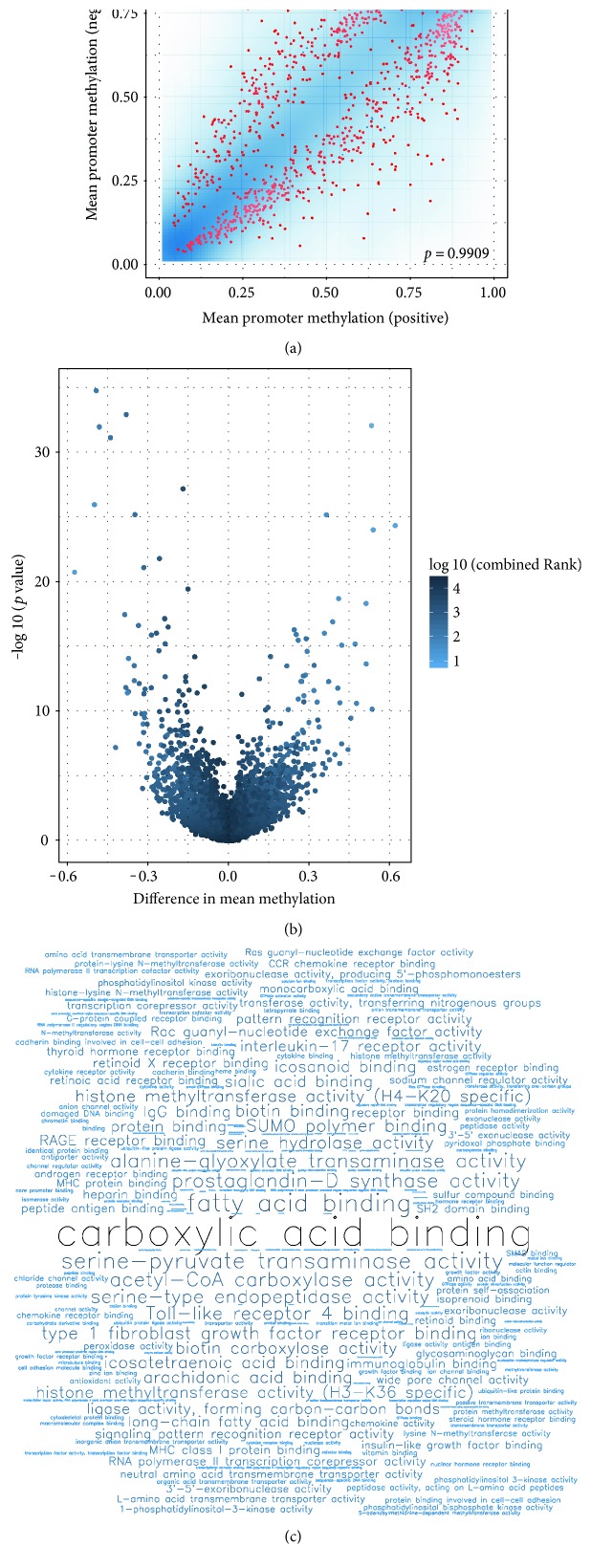
Differential methylation of samples based on traits and GO enrichment. (a) Density plot of average promoter methylation in T (15; 17) positive (*x*-axis) versus negative (*y*-axis) patient groups. The density plot is overlaid with a scatter plot in which promoters with FDR-corrected *p* value < 0.05 are depicted as red points. (b) Volcano plot for T (15; 17) showing the relationship between differences in group mean methylation and uncorrected *p* value for every gene promoter. (c) Word cloud showing the most enriched Gene Ontology terms in the category molecular function, when the top 100 hypomethylated gene promoters in the T (15; 17)-positive patient group are considered. Larger font size indicates a more significant *p* value.

Differential methylation on CpG sites and gene promoters was computed using different metrics, including limma statistical test. The differentially methylated promoters for T (15; 17) are depicted as red points in the scatter plot of Figure 5(a). Despite the overall very high correlation of average promoter methylation between the T (15; 17)-positive and T (15; 17)-negative patient groups, there is a substantial number of gene promoters for which the two groups show a significant difference in methylation (mean beta value). The volcano plot in Figure 5(b) shows the relationship between mean methylation difference and the *p* values obtained from the limma test. Word clouds for Gene Ontology (GO) enrichment analysis of T (15; 17) indicate aberrations in the carboxylic acid-binding pathway based on promoter hypomethylation (Figure 5(c)).

### 3.8. Highly Methylated Genes in AML

**Figure 6:**
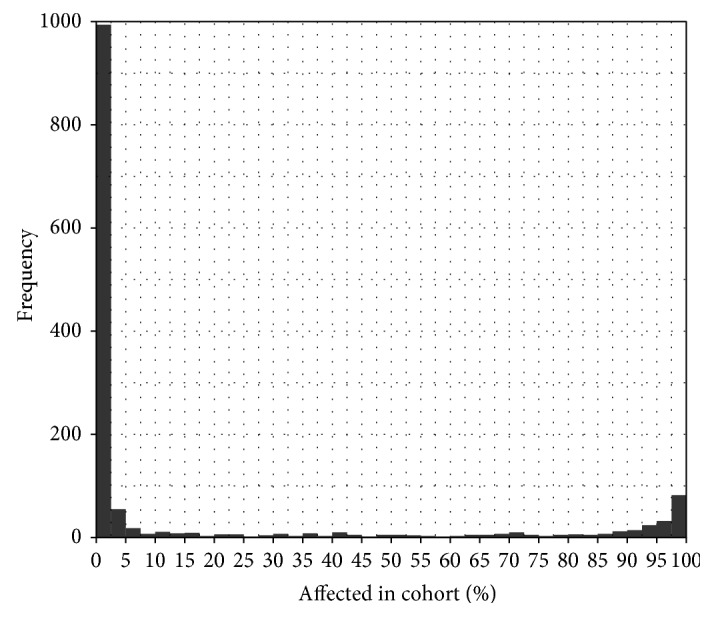
Highly methylated gene promoters in the dataset. Almost 200 gene promoters are highly methylated in 90% of the AML patients. We checked the distribution of those genes across the chromosomes in Figure 7.

**Figure 7:**
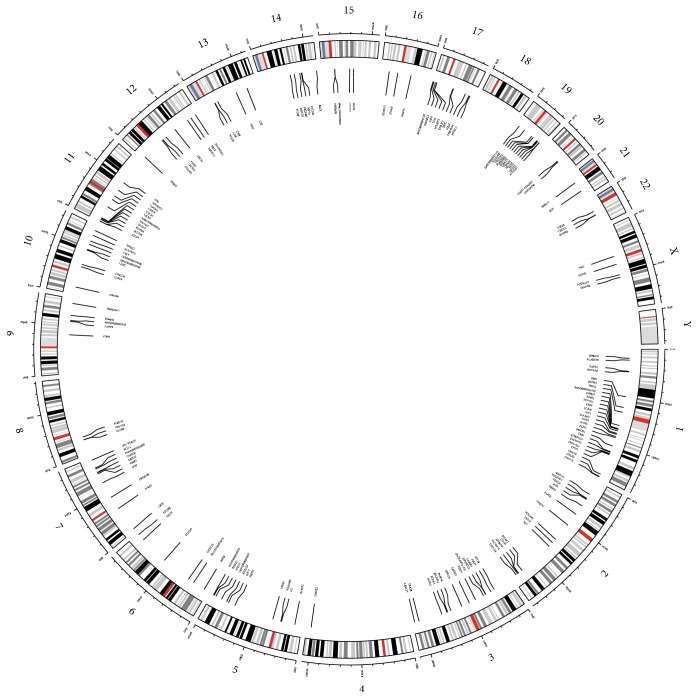
Distribution of hypermethylated genes in chromosomes. Commonly hypermethylated genes in AML are randomly distributed across the chromosomes. Chromosome 1 contains the highest number of genes than any other chromosomes.

We found more than 1300 highly methylated gene promoters in the AML dataset. Around 1100 genes were hypermethylated for only 5% patients. There are 200 gene promoters that were hypermethylated among 90% patients (Figure 6). These 200 genes were one of our areas of interest, whether their distribution in the chromosome follows any pattern. We generated a circos plot for those frequently (>90%) hypermethylated genes with associated chromosomal position (Figure 7). There was no particular distribution pattern of those genes across chromosomes; with the notable exception of Y and 18. Chromosome 1 showed a comparatively large number of hypermethylated genes compared to other chromosomes.

### 3.9. Gene Mutation and Hypermethylation Pattern

**Figure 8:**
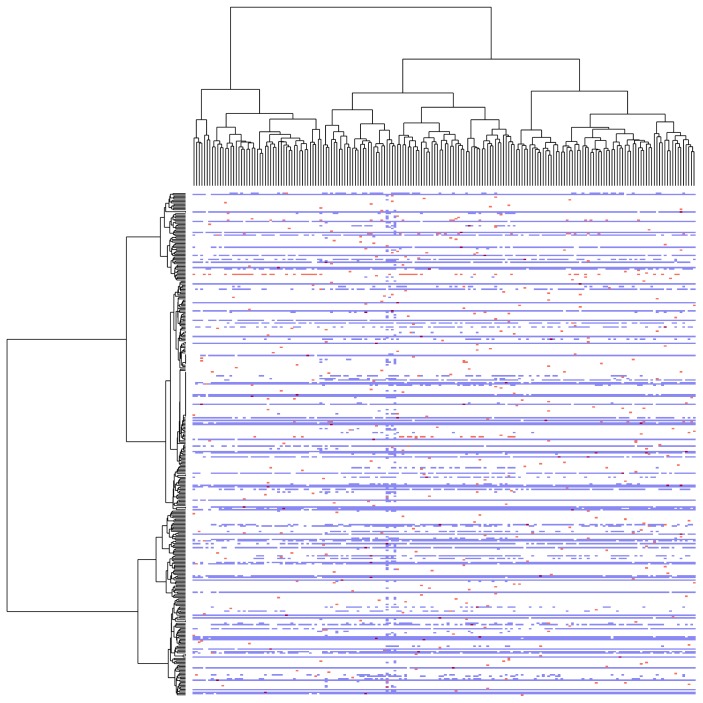
Pattern recognition after combination of methylation and mutation. *x*-axis represents the number of patients (*n* = 200), and *y*-axis represents the number of the most hypermethylated genes (*n* = 879). Dendrogram represents three clusters of AML patients and four clusters of the most hypermethylated genes.

Only a small number of AML patients show high mutation, and majority patients are without any known driver gene mutation (Figure S5). We plotted all highly mutated and hypermethylated genes against 200 AML cases in a single heatmap (Figure 8). Genes that were both mutated and hypermethylated were indicated with different color signs. Mutation or hypermethylation was also shown in different colors. Unsupervised hierarchical clustering dendrogram uncovered some interesting clusters. There were three groups of patients and four major groups of genes clearly visible. Genes that are both mutated and hypermethylated were scattered randomly. This is quite an interesting pattern that could be useful in the future for better AML classification. The specialties among those groups are still unknown and would be a future area of our interest.

### 3.10. Pathway Analysis

Integrated pathway analysis for AML was performed using IPA online database, and deregulated transcripts are listed in Table S1. The total number of factors (genes, transcription factors, and RNAs) affected was 1083 (checked on September 23, 2015). Upregulated transcripts were numbered 64 and downregulated transcripts were numbered 29. Proteasome subunit beta type-9 (PSMB9) and other PSMBs were downregulated and hampered assembly of the proteasome complex in AML. Only 15 miRNAs were found directly affected in the case of AML, and miR-10 was affected in multiple AML subtypes.

### 3.11. Survival Curves

**Figure 9:**
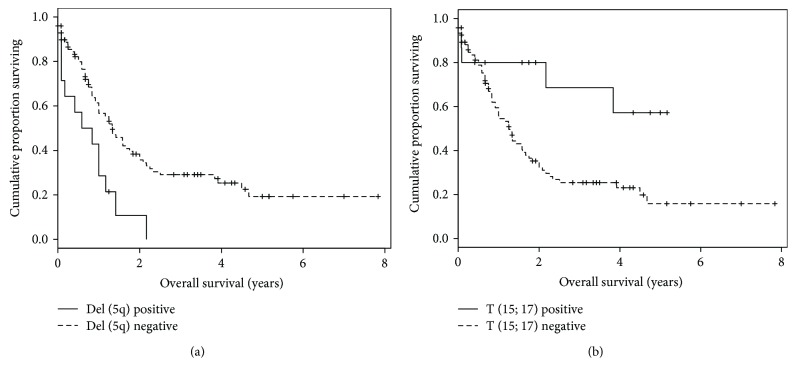
Kaplan-Meier survival curves. The *x*-axis lists survival time in years, and the *y*-axis represents cumulative survival proportion. (a) The survival time of 50% of the Del (5q)-positive patients is less than 1 year. (b) The survival time of 50% of the T (15; 17)-positive patients is more than 4 years.

We combined all risk factors and traits in order to draw Kaplan-Meier survival curves (Figure 9). We calculated *p* values for all possible traits (Table S2), and only Del (5q), T (9; 11), T (9; 22), and T (15; 17) showed statistical significance (0.0149, 0.0233, 0.0443, and 0.0457, resp.) after necessary adjustment with the age and sex of the patients. Patients with Del (5q) positive and T (15; 17) positive showed distinctive results. The Del (5q)-positive patients' average survival rate was less than 1 year (Figure 9(a)). But the T (15; 17)-positive patients' average survival rate was more than 3 years (Figure 9(b)). Other traits did not affect much in the case of patient survival, and also, most of them were not statistically significant. Only T (15; 17) showed a higher survival rate, and the causes were unknown. We hypothesized that maybe some chemotherapeutic drugs were working better due to this particular translocation, and those patients survived more than others.

## 4. Discussion

AML is a complex genetic disease because of the accumulation of numerous genomic lesions that regulate the expression of oncogenes as well as tumor-suppressor genes. Chromosomal aberrations that cause gene fusions were mostly considered as markers for diagnosis, prognosis, and classification in the early days [29–31]. There were some problems with classification based on cytogenetic factors, because AML patients with normal karyotypes (CN-AML) were classified as the intermediate risk group [32]. Although this heterogeneous group was further classified based on genetic factors, many cases had been reported without even genetic abnormalities. The incorporation of epigenetic factors with genetic and cytogenetic factors might be helpful for better classification as well as diagnostic and prognostic risk stratification.

The classification of AML considering all possible genetic, epigenetic, and cytogenetic aberrations is not only a complex and challenging problem but also a matter of clinical validation that was never done before. Here, we integrated all available data from TCGA and tried to look for any pattern that could be useful for patient classification. We used different software and strategies to visualize the dataset. This comprehensive and large-scale study of mutation and methylation in human disease demonstrates that genetic and epigenetic patterns within the biological and clinical signatures and DNA methylation classifiers can be derived from population studies with clinical predictive capacity [33]. Although we have not attempted to subclassify AML in this study, some identified pattern (Figure 8) would help to better understand the overall data structure through which clinically implementable classification might become possible in the future. Our analyses demonstrate that systematic integration of gene expression and DNA methylation profile could improve the classification methods. CN-AML especially could be understood better and in broader ways.

Although the vast majority of regulatory elements are located outside promoters, most of the DNA methylation studies in AML have focused mainly on CGI promoter regions [34, 35]. There are almost 28 million CpG dinucleotides in the human genome [36], and only 2% of them are located within gene-promoter regions [13]. Significant statistical and biological evidence of non-CGI methylation is now increasing and suggests it plays an important role in the regulation of gene expression [37, 38]. In our study, we found that total numbers of methylation distributions are high at open sea and shore (Figure 3(b)) compared to those of promoter regions. In CLL, methylation alterations have been reported in non-CGI regions [39]. DNA methylation in shores is strongly correlated with gene expression changes in colorectal cancer [40, 41]. Non-CGI DNA methylation can be used as a biomarker for the differentiation of pluripotent stem cells [42]. Leukemogenic mutations alone, such as FLT3, NPM1, CEBPA, and DNMT3A, are not sufficient to explain diverse clinical AML subtypes; epigenetic alterations are abundant and common and might explain the biology behind various AML groups.

It has been reported that some genes (FLT3, NPM1, and DNMT3A) are recurrently mutated (more than 20%) in AML [11]. But it is not clear yet which genes are particularly responsible for that specific methylation pattern. The mutation of NPM1 was associated with four slightly distinct epigenetic signatures that cannot be explained by concurrent FLT3-ITD mutation [33]. This suggests that there might be some unrecognized mechanisms to determine different epigenetic groups. Our study also suggests that almost 200 genes are recurrently hypermethylated (Figure 6) in 90% of AML cases and evenly distributed among chromosomes (Figure 7).

As DNA methyltransferases are responsible for de novo methylation, DNMT3A is a crucial gene for the epigenetic alteration of AML. DNMT3A-mutated patients show global hypomethylation [13], because of recently reported impaired catalytic activity due to this mutation [43]. There are different patterns of DNMT3A methylation in AML cases. In bone marrow, mononucleotide cells in AML showed significant hypomethylation of DNMT3A [44], whereas in peripheral blood cells, this gene was found hypermethylated [45]. We can say that aberrant DNMT3A methylation would be an independent negative prognostic factor in AML. In our analysis, many traits have been associated with abnormal methylation patterns. T (15; 17)-positive patients showed global hypomethylation, and Del (5q) correlates with hypermethylation (Figure 4). Other traits not showing any significant cluster or pattern in a heatmap might still be informative but not detected due to insufficient sample numbers or molecular heterogeneity between inter- and intratraits.

One of the notable findings of this study is the identification of clusters in a heatmap (Figure 8) with the combination of all probable genetic and epigenetic signatures. Three groups of patients are clearly visible through the *x*-axis of the figure, whereas four groups of genes are able to be identified through *y*-axis. Genes that are both methylated and mutated have no clear pattern. We are not able to figure out yet the common or special characteristics of those clusters. This could be a further area of study before considering epigenetic patterns as factors for AML patient classification in clinics.

Another important observation in AML dataset is the role of traits for patient survival. Del (5q)-positive patients survive less than one year. Only T (15; 17) is associated with an exceptionally higher survival rate (>3 years) compared to other traits (Figure 9(b)). We hypothesize that maybe some chemotherapeutic drugs were more effective due to this translocation. There is no prescribed drug information in the TCGA AML clinical data files. It is known that many drugs were designed by targeting this particular translocation which may influence this high survival rate. A large cohort of AML patients with detailed clinical information is required to validate the hypothesis. Survival curves (Kaplan-Meier) for many traits were not statistically significant (0.05) due to small positive sample size, and some traits (like FLT3 and NPMc) showed the same survival pattern for both positive and negative patients [46].

There are some drawbacks of this study that should be considered before clinical implementation. Samples were collected over a long period of time, and all clinical tests were not performed equally. Many routine tests changed in the clinic, and new genetic testing was assigned. Some data were missing in the clinical data files. Feasibility of the findings has not been tested in the clinical context. Although we presented statistically significant data, statistical significance might not always be significant clinically.

Finally, this secondary data analysis revealed global pictures of AML genomes that would be useful for further broad spectrum analysis and interpretation of cancer data. Our integrated analysis approaches show some interesting findings like epigenetic patterns and survival curves. We show how individual traits are associated with global methylation changes. In conclusion, a combination of genetic, epigenetic, and cytogenetic data could further expand our understanding of the biology of AML. This analysis would motivate to other groups to increase the sample size to confirm our findings as well as to include epigenetic signatures for the classification of AML. We hope our analysis might motivate clinicians to eventually include epigenetic signatures in the classification of AML and inspire to investigate patient survival, T (15; 17), and particular drug responses.

## Supplementary Material

Differential Analysis of Genetic, Epigenetic, and Cytogenetic Abnormalities in AML

## Figures and Tables

**Table 1:** Characteristics of AML patients.

Patient characteristics	Values (units)
Gender (M/F)	109/91
Age at diagnosis	55.50 ± 16.07 (years)
Race (black/white/NA)	15/183/2
Ethnicity (nonhispanic/hispanic/NA)	194/3/3
Vital status (dead/alive)	133/67
Blasts in peripheral blood (M/F)^1^	67.76/64.86 (%)
WBC counts after 24 h of storage^2^	37.246 (k/mm^3^)
Platelets counts	65.98 (cells/*μ*L)
Blast cell counts	36.28 (cells/*μ*L)
Neutrophil counts after 24 h of storage	12.16 (k/mm^3^)
Lymphocytes counts	27.50 (cells/*μ*L)
Monocytes counts	12.34 (cells/*μ*L)
Number of patients with cytogenetic abnormalities	106
Number of patients with genetic abnormalities	160

M: male; F: female; NA: not available. ^1^Blasts = immature white blood cells. More than 20% of blasts is generally required for a diagnosis of AML. ^2^Number of WBCs reduce after long time storage.

## References

[1] Tenen D. G. (2003). Disruption of differentiation in human cancer: AML shows the way. *Nature Reviews Cancer*.

[2] Mehdipour P., Santoro F., Minucci S. (2015). Epigenetic alterations in acute myeloid leukemias. *FEBS Journal*.

[3] Siegel R., Ma J., Zou Z., Jemal A. (2014). Cancer statistics, 2014. *CA: A Cancer Journal for Clinicians*.

[4] Byrd J. C., Mrózek K., Dodge R. K. (2002). Pretreatment cytogenetic abnormalities are predictive of induction success, cumulative incidence of relapse, and overall survival in adult patients with de novo acute myeloid leukemia: results from Cancer and Leukemia Group B (CALGB 8461). *Blood*.

[5] Christopeit M., Bartholdy B. (2014). Epigenetic signatures as prognostic tools in acute myeloid leukemia and myelodysplastic syndromes. *Epigenomics*.

[6] Vardiman J. W., Thiele J., Arber D. A. (2009). The 2008 revision of the World Health Organization (WHO) classification of myeloid neoplasms and acute leukemia: rationale and important changes. *Blood*.

[7] Patel J. P., Gönen M., Figueroa M. E. (2012). Prognostic relevance of integrated genetic profiling in acute myeloid leukemia. *New England Journal of Medicine*.

[8] Shen Y., Zhu Y. M., Fan X. (2011). Gene mutation patterns and their prognostic impact in a cohort of 1185 patients with acute myeloid leukemia. *Blood*.

[9] Bock C., Beerman I., Lien W. H. (2012). DNA methylation dynamics during in vivo differentiation of blood and skin stem cells. *Molecular Cell*.

[10] Ji H., Ehrlich L. I., Seita J. (2010). Comprehensive methylome map of lineage commitment from haematopoietic progenitors. *Nature*.

[11] Network CGAR (2013). Genomic and epigenomic landscapes of adult de novo acute myeloid leukemia. *The New England Journal of Medicine*.

[12] Lokody I. (2014). Epigenetics: histone methyltransferase mutations promote leukaemia. *Nature Reviews Cancer*.

[13] Qu Y., Lennartsson A., Gaidzik V. I. (2014). Differential methylation in CN-AML preferentially targets non-CGI regions and is dictated by DNMT3A mutational status and associated with predominant hypomethylation of HOX genes. *Epigenetics*.

[14] Delhommeau F., Dupont S., Della Valle V. (2009). Mutation in TET2 in myeloid cancers. *New England Journal of Medicine*.

[15] Michaux L., Wlodarska I., Stul M. (2000). MLL amplification in myeloid leukemias: a study of 14 cases with multiple copies of 11q23. *Genes, Chromosomes and Cancer*.

[16] Figueroa M. E., Abdel-Wahab O., Lu C. (2010). Leukemic IDH1 and IDH2 mutations result in a hypermethylation phenotype, disrupt TET2 function, and impair hematopoietic differentiation. *Cancer Cell*.

[17] Mendler J. H., Maharry K., Radmacher M. D. (2012). RUNX1 mutations are associated with poor outcome in younger and older patients with cytogenetically normal acute myeloid leukemia and with distinct gene and MicroRNA expression signatures. *Journal of Clinical Oncology*.

[18] Chou W. C., Huang H. H., Hou H. A. (2010). Distinct clinical and biological features of de novo acute myeloid leukemia with additional sex comb-like 1 (ASXL1) mutations. *Blood*.

[19] Eppert K., Takenaka K., Lechman E. R. (2011). Stem cell gene expression programs influence clinical outcome in human leukemia. *Nature Medicine*.

[20] Schoch C., Kohlmann A., Schnittger S. (2002). Acute myeloid leukemias with reciprocal rearrangements can be distinguished by specific gene expression profiles. *Proceedings of the National Academy of Sciences*.

[21] Gentles A. J., Plevritis S. K., Majeti R., Alizadeh A. A. (2010). Association of a leukemic stem cell gene expression signature with clinical outcomes in acute myeloid leukemia. *Jama*.

[22] Christopeit M., Kröger N., Haferlach T., Bacher U. (2014). Relapse assessment following allogeneic SCT in patients with MDS and AML. *Annals of Hematology*.

[23] Bennett J. M., Catovsky D., Daniel M. T. (1976). Proposals for the classification of the acute leukaemias. French-American-British (FAB) co-operative group. *British Journal of Haematology*.

[24] Vardiman J. W., Harris N. L., Brunning R. D. (2002). The World Health Organization (WHO) classification of the myeloid neoplasms. *Blood*.

[25] Weinstein J. N., Cancer Genome Atlas Research Network, Collisson E. A. (2013). The cancer genome atlas pan-cancer analysis project. *Nature Genetics*.

[26] Assenov Y., Müller F., Lutsik P., Walter J., Lengauer T., Bock C. (2014). Comprehensive analysis of DNA methylation data with RnBeads. *Nature Methods*.

[27] Bock C. (2012). Analysing and interpreting DNA methylation data. *Nature Reviews Genetics*.

[28] Bock C., Walter J., Paulsen M., Lengauer T. (2008). Inter-individual variation of DNA methylation and its implications for large-scale epigenome mapping. *Nucleic Acids Research*.

[29] Grimwade D., Hills R. K., Moorman A. V. (2010). Refinement of cytogenetic classification in acute myeloid leukemia: determination of prognostic significance of rare recurring chromosomal abnormalities among 5876 younger adult patients treated in the United Kingdom Medical Research Council trials. *Blood*.

[30] Hehlmann R., Grimwade D., Simonsson B. (2010). The European LeukemiaNet-Achievements and perspectives. *Haematologica*.

[31] Marcucci G., Haferlach T., Döhner H. (2011). Molecular genetics of adult acute myeloid leukemia: prognostic and therapeutic implications. *Journal of Clinical Oncology*.

[32] Lahortiga I., Cools J. (2012). New Opportunities and New Problems for Acute Myeloid Leukemia Treatment. *Haematologica*.

[33] Figueroa M. E., Lugthart S., Li Y. (2010). DNA methylation signatures identify biologically distinct subtypes in acute myeloid leukemia. *Cancer Cell*.

[34] Deneberg S., Grövdal M., Karimi M. (2010). Gene-specific and global methylation patterns predict outcome in patients with acute myeloid leukemia. *Leukemia*.

[35] Yalcin A., Kreutz C., Pfeifer D. (2013). MeDIP coupled with a promoter tiling array as a platform to investigate global DNA methylation patterns in AML cells. *Leukemia Research*.

[36] Suzuki M. M., Bird A. (2008). DNA methylation landscapes: provocative insights from epigenomics. *Nature Reviews Genetics*.

[37] De S., Shaknovich R., Riester M. (2013). Aberration in DNA methylation in B-cell lymphomas has a complex origin and increases with disease severity. *PLoS Genetics*.

[38] Suzuki M., Oda M., Ramos M. P. (2011). Late-replicating heterochromatin is characterized by decreased cytosine methylation in the human genome. *Genome Research*.

[39] Cahill N., Bergh A. C., Kanduri M. (2013). 450K-array analysis of chronic lymphocytic leukemia cells reveals global DNA methylation to be relatively stable over time and similar in resting and proliferative compartments. *Leukemia*.

[40] Irizarry R. A., Ladd-Acosta C., Wen B. (2009). The human colon cancer methylome shows similar hypo-and hypermethylation at conserved tissue-specific CpG island shores. *Nature Genetics*.

[41] Akalin A., Garrett-Bakelman F. E., Kormaksson M. (2012). Base-pair resolution DNA methylation sequencing reveals profoundly divergent epigenetic landscapes in acute myeloid leukemia. *PLoS Genetics*.

[42] Butcher L. M., Ito M., Brimpari M. (2016). Non-CG DNA methylation is a biomarker for assessing endodermal differentiation capacity in pluripotent stem cells. *Nature Communications*.

[43] Holz-Schietinger C., Matje D. M., Reich N. O. (2012). Mutations in DNA methyltransferase (DNMT3A) observed in acute myeloid leukemia patients disrupt processive methylation. *Journal of Biological Chemistry*.

[44] Zhang Y. Y., Yao D. M., Zhu X. W. (2015). DNMT3A intragenic hypomethylation is associated with adverse prognosis in acute myeloid leukemia. *Leukemia Research*.

[45] Jost E., Lin Q., Weidner C. I. (2014). Epimutations mimic genomic mutations of DNMT3A in acute myeloid leukemia. *Leukemia*.

[46] Islam M. M., Mohamed Z., Assenov Y. Differential analysis of genetic, epigenetic, and cytogenetic abnormalities in AML.

