# Supplementary material for: Differential Analysis of Genetic, Epigenetic, and Cytogenetic Abnormalities in AML
Source: Int J Genomics. 2017 Jun 20;2017:2913648. doi: 10.1155/2017/2913648 (PMC5496127; doi:10.1155/2017/2913648)
Supplement: Supplementary file 1 — Differential Analysis of Genetic, Epigenetic, and Cytogenetic Abnormalities in AML [file 2913648.f1.docx]

**Differential Analysis of Genetic, Epigenetic, and Cytogenetic Abnormalities in AML**

Mirazul Islam, Zahurin Mohamed, and Yassen Assenov.

**Supplemental File**

**Table S1: List of AML associated deregulated transcripts.***

| Symbol | Molecule Type | Expression evidence |
| --- | --- | --- |
| 26s Proteasome | **complex** | **upregulation** |
| AURKA | **kinase** | **upregulation** |
| BCL2L13 | **other** | **upregulation** |
| CD86 | **transmembrane receptor** | **upregulation** |
| CSF2RB | **transmembrane receptor** | **upregulation** |
| E2F1 | **transcription regulator** | **upregulation** |
| E2F3 | **transcription regulator** | **upregulation** |
| E4F1 | **transcription regulator** | **upregulation** |
| EEF1A1 | **translation regulator** | **upregulation** |
| EHMT2 | **transcription regulator** | **upregulation** |
| F3 | **transmembrane receptor** | **upregulation** |
| FOXM1 | **transcription regulator** | **upregulation** |
| GNB2L1 | **enzyme** | **upregulation** |
| GPX3 | **enzyme** | **upregulation** |
| HLA-DRA | **transmembrane receptor** | **upregulation** |
| IL1B | **cytokine** | **upregulation** |
| IL3RA | **transmembrane receptor** | **upregulation** |
| let-7 | **microRNA** | **upregulation** |
| LYZ | **enzyme** | **upregulation** |
| MDM2 | **transcription regulator** | **upregulation** |
| mir-10 | **microRNA** | **upregulation** |
| miR-10a-5p | **mature microRNA** | **upregulation** |
| mir-129 | **microRNA** | **upregulation** |
| miR-129-5p | **mature microRNA** | **upregulation** |
| mir-155 | **microRNA** | **upregulation** |
| miR-155-5p | **mature microRNA** | **upregulation** |
| mir-17 | **microRNA** | **upregulation** |
| miR-17-5p | **mature microRNA** | **upregulation** |
| mir-181 | **microRNA** | **upregulation** |
| miR-181a-5p | **mature microRNA** | **upregulation** |
| mir-320 | **microRNA** | **upregulation** |
| miR-320b | **mature microRNA** | **upregulation** |
| MT-CO2 | **enzyme** | **upregulation** |
| MT-CO3 | **enzyme** | **upregulation** |
| MT-ND4 | **enzyme** | **upregulation** |
| MYB | **transcription regulator** | **upregulation** |
| MYCN | **transcription regulator** | **upregulation** |
| NCAM1 | **other** | **upregulation** |
| PDHA1 | **enzyme** | **upregulation** |
| PDP1 | **phosphatase** | **upregulation** |
| PDPK1 | **kinase** | **upregulation** |
| PPP1R13L | **transcription regulator** | **upregulation** |
| PSIP1 | **other** | **upregulation** |
| PSME1 | **other** | **upregulation** |
| PTPN6 | **phosphatase** | **upregulation** |
| RPL11 | **other** | **upregulation** |
| RPL3 | **other** | **upregulation** |
| RPL34 | **other** | **upregulation** |
| RPL41 | **other** | **upregulation** |
| RPL6 | **other** | **upregulation** |
| RPS12 | **other** | **upregulation** |
| RPS24 | **other** | **upregulation** |
| RPS25 | **other** | **upregulation** |
| RPS4X | **other** | **upregulation** |
| RUNX1 | **transcription regulator** | **upregulation** |
| SALL4 | **transcription regulator** | **upregulation** |
| SERPINB2 | **other** | **upregulation** |
| STAT5B | **transcription regulator** | **upregulation** |
| TET1 | **other** | **upregulation** |
| TP53 | **transcription regulator** | **upregulation** |
| UBE2I | **enzyme** | **upregulation** |
| WT1 | **transcription regulator** | **upregulation** |
| XIAP | **enzyme** | **upregulation** |
| ZFP36L2 | **transcription regulator** | **upregulation** |
| APH1A | **peptidase** | **downregulation** |
| CTBP2 | **transcription regulator** | **downregulation** |
| DLL1 | **enzyme** | **downregulation** |
| DLL4 | **other** | **downregulation** |
| DTX1 | **transcription regulator** | **downregulation** |
| DTX3 | **other** | **downregulation** |
| DVL3 | **other** | **downregulation** |
| FBXL2 | **enzyme** | **downregulation** |
| GATA3 | **transcription regulator** | **downregulation** |
| HES1 | **transcription regulator** | **downregulation** |
| HES5 | **other** | **downregulation** |
| HEY1 | **transcription regulator** | **downregulation** |
| HEY2 | **transcription regulator** | **downregulation** |
| HEYL | **transcription regulator** | **downregulation** |
| HK3 | **kinase** | **downregulation** |
| INCA1 | **other** | **downregulation** |
| KAT5 | **transcription regulator** | **downregulation** |
| LYN | **kinase** | **downregulation** |
| mir-34 | **microRNA** | **downregulation** |
| miR-34a-5p | **mature microRNA** | **downregulation** |
| NOTCH1 | **transcription regulator** | **downregulation** |
| NRARP | **transcription regulator** | **downregulation** |
| PRSS1 | **peptidase** | **downregulation** |
| PRSS2 | **peptidase** | **downregulation** |
| PSEN1 | **peptidase** | **downregulation** |
| PSEN2 | **peptidase** | **downregulation** |
| PSMB10 | **peptidase** | **downregulation** |
| PSMB8 | **peptidase** | **downregulation** |
| PSMB9 | **peptidase** | **downregulation** |

* Information collected from IPA (Ingenuity pathway analysis).

**Table S2: Statistical significance of survival curves.**

| Aberration | Positive | Negative | P-value (adjusted) |
| --- | --- | --- | --- |
| Inv (16) | 9 | 103 | 0.1671 |
| Del (5q) | 15 | 103 | **0.0149*** |
| Del (7q) | 21 | 102 | 0.0656 |
| T (4;11) | 1 | 103 | 0.8124 |
| T (8;21) | 7 | 103 | 0.3686 |
| T (9;11) | 2 | 103 | **0.0233*** |
| T (9;22) | 3 | 103 | **0.0443*** |
| T (15;17) | 16 | 98 | **0.0457*** |
| Trisomy 21 | 8 | 103 | 0.1900 |
| Trisomy 8 | 20 | 101 | 0.7451 |
| Activating RAS | 11 | 180 | 0.7481 |
| BCR-ABL | 1 | 15 | n/a |
| FLT3 Mutation | 57 | 130 | 0.0744 |
| IDH1 R132 | 18 | 171 | 0.9118 |
| IDH1 R140 | 15 | 175 | 0.2802 |
| IDH1 R172 | 2 | 188 | 0.3275 |
| NPMc | 45 | 145 | 0.2866 |
| PML-RAR | 8 | 9 | 0.0675 |
| Cytogenetically normal | 97 | 97 | 0.9888 |

* Significant p-value

**Figure S1: Summary of patient’s clinical tests.**

X-axis represents the number of patients and Y-axis represents the clinical information. Different clinical observations are shown in the plot. No epigenetic testing performed in the clinic. Cytogenetic test and Fluorescence in situ hybridization (FISH) test are performed for most of the patient cohort. ‘History of other malignancy’ means malignancy other than haematological malignancy. One fourth of the total patients received neoadjuvant treatment and only handful number of patients show haematological disorder other that AML.


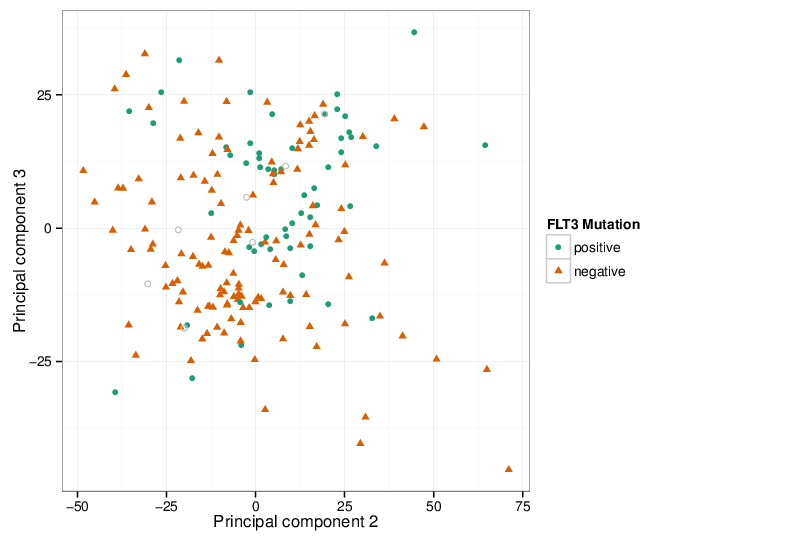


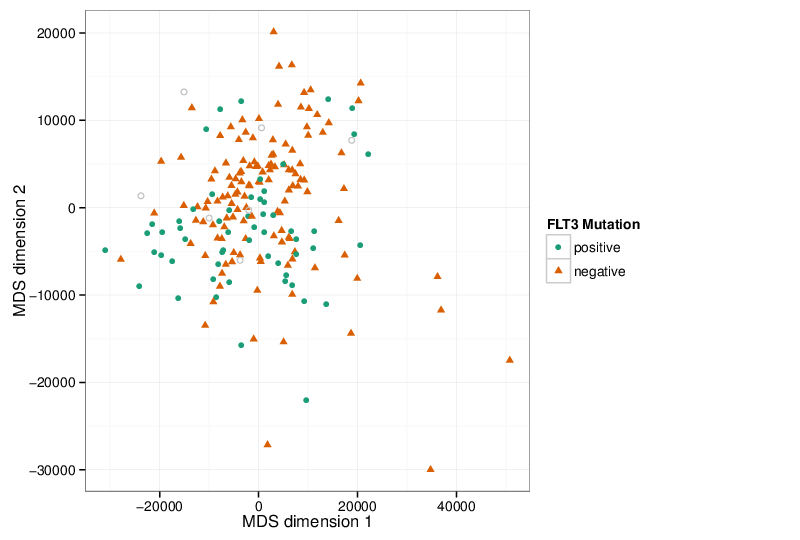


**Figure S2: Low-dimensional representation of AML dataset.**

(a) Principal component (PC) 1 is not significant for FLT3. Although PC2 and PC3 are significant (Figure S2) for FLT3, there is no good separation for positive and negative patients in principal component analysis. Gray circle represent missing values. (b) Multidimensional scaling (MDS) for FLT3 after performing Kruskal's non-metric multidimensional scaling test.

 Wilcoxon rank sum test performed for first eight principal components (PC) across all the traits that together can explain more than 99% of total variance across the patient cohort. Significant p-values (less than 0.01) are printed in pink boxes. Non-significant values are represented by blue boxes. PC1 is not significant for most of the traits except IDH1 R140. We used PC2 and PC3 for scatter plot in Figure 2 and S3 that together can explain the highest variances across the patient cohort. PC3 is highly significant for NPMc (p=5.1E-13) and FLT3 (p=2.7E-4).


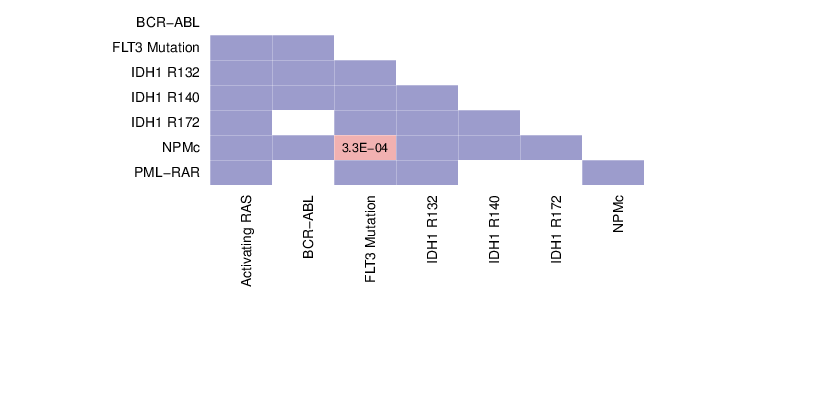


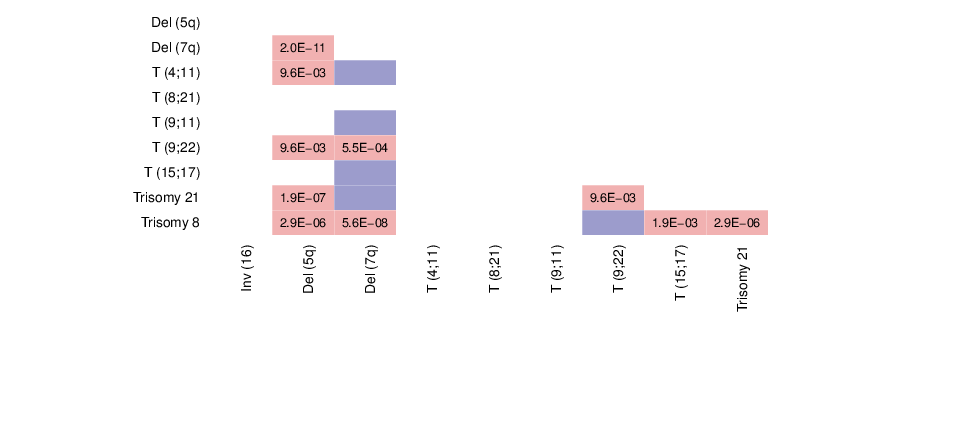


**Figure S4: Associations between pairs of traits.**

Fisher’s exact test was performed for significant association. (a) FLT3 and NPMc are significantly correlated (Kruskal-Wallis one-way analysis of variance). (b) Del (5q) is correlated for most of the cytogenetic traits whereas blank box represent unable to perform test due to small sample size.


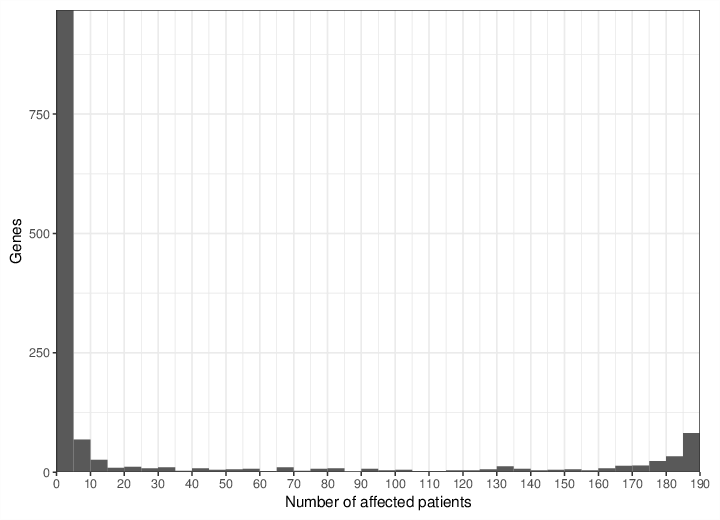


**Figure S5: Distribution of mutated genes in the dataset.**

Only a small number of AML patients show high mutation and majority patients are without any mutation in known driver genes.
